# What Is the Value of Surgical Intervention for Sacral Metastases?

**DOI:** 10.1371/journal.pone.0168313

**Published:** 2016-12-19

**Authors:** Zhiye Du, Wei Guo, Rongli Yang, Xiaodong Tang, Tao Ji, Dasen Li

**Affiliations:** Department of Musculoskeletal Tumor Centre, Peking University &People's Hospital, Beijing, China; Kanazawa University, JAPAN

## Abstract

**Objective:**

To investigate the impact of surgery on local control and quality of life for patients with sacral metastases and to determine whether the complications of surgery were acceptable.

**Methods:**

Curettage for metastatic lesions of the sacrum was performed in 154 patients with obvious sacral nerve compression symptoms between July 1997 and July 2014. Potential risk factors were evaluated in univariate analysis for associations with local control; multivariate conditional logistic regression was used to identify the combined effects. Complications were recorded. The pre- and postoperative visual analogue scale of pain, Eastern Cooperative Oncology Group (ECOG) scores, and quality of life scores were collected to evaluate the impact of surgery.

**Results:**

The cumulative survival rates were 71.8%, 41.1%, and 22.5% and the local control rates were 95.4%, 90.9%, and 79.4% at 6, 12, and 24 months, respectively. Tumors with rapid growth, the lack of preoperative radiotherapy, and application of aortic balloon occlusion were significantly associated with good local control. There were 29 (18.8%) complications related to surgery. The mean pain scores were 7.04 preoperatively, 1.66 at 1 month postoperatively (*p* = 0.003), and 1.51 at 3 months postoperatively (*p* = 0.002). The mean ECOG scores were 2.82 preoperatively and 1.47 3 months postoperatively (*p* < 0.001). There were significant improvements from preoperatively to 3 months postoperatively in global health status (43.3 vs. 52.1), pain (62.0 vs. 33.2), and constipation (51.4 vs. 30.3) (*p* < 0.001).

**Conclusions:**

Surgery for sacral metastasis is effective to palliate pain rapidly and improve constipation and quality of life and has a low rate of complications.

## Introduction

Tumors of the sacrum are rare; including primary and metastatic lesions [1, 2], they account for 1–7% of all spinal tumors that come to clinical attention [3]. Metastatic tumors represent the majority of malignant sacral tumors. With improved treatments for cancer, the number of patients with bony metastasis has increased and their survival time has lengthened [4]. Metastatic bone disease affecting the sacrum is increasingly encountered by oncologists and orthopedic surgeons [5].

Traditional conservative radiotherapy has long been the preferred first-line intervention for sacral metastasis. However, as the survival time of patients has lengthened, failure of local control has occurred in more patients who undergo traditional radiotherapy. In this situation, surgery could be considered as an important palliative approach for selected candidates with severe disease. In addition, surgical intervention for metastatic sacral tumors used to be technically difficult, challenging, and risky. However, as surgical technique has improved and the technique of aortic balloon occlusion has been applied, the intraoperative and postoperative blood loss has decreased enormously, which has greatly reduced the risk of surgery [6]. However, very few studies that included sufficient patient numbers and types of pathology have reported the results of surgical intervention for sacral metastasis [7, 8]. The effects and complications of surgery for sacral metastases remain unclear.

The goal of the present study was to investigate the impact of surgery on local control, improvement of visual analogue scale (VAS) pain scores, performance status evaluated by Eastern Cooperative Oncology Group (ECOG) scores, and quality of life (QoL) evaluated by QLQ-C30 scoring system for patients with sacral metastases, and whether the complications of surgery were acceptable.

## Materials and Methods

The Ethics Committee of Peking University People’s Hospital, China approved this retrospective study. Written informed consent to participate in this study was obtained from all patients. Each author certifies that his or her institution has approved the reporting of these cases and that all investigations were conducted in conformity with the ethical principles of research. The records of 180 patients with sacral metastases who were admitted to the Musculoskeletal Tumor Center of Peking University People’s Hospital between July 1997 and June 2015 were reviewed retrospectively. All patients received pre- or postoperative systematic treatment. The surgical indications included intractable pain unresponsive to conservative therapy (acesodyne therapy, radiotherapy, and/or chemotherapy), neurological deficit, instability of sacroiliac articulation, Tomita score ≤ 7, estimated survival time > 3 months, and the patient’s persistent desire for surgery. The inclusion criteria included diagnosed metastatic sacral tumor. Patients received the surgical intervention and complete follow-up for at least 3 months. We excluded 10 patients who refused to undergo surgery, eight patients who were lost to follow-up, six patients who received *en bloc* resection with lesions located below S3 and two patients who died early postoperatively from complications not associated with the tumors. Thus, 154 patients remained and were reviewed retrospectively (Table 1).

**Table 1:** Demographics and Surgical Details of the 154 Patients Included in this Study.

Details	n	Percent
Sex		
Male	87	56.5%
Female	67	43.5%
Age		
< 60	92	59.7%
≥ 60	62	40.4%
Surgical approach		
Posterior only	145	94.2%
Combined anterior/posterior	9	5.8%
Metastatic lesion involving level		
S1	145	94.2%
S2	99	64.3%
S3	30	19.5%
S4	11	7.1%
Sacroiliac junction	105	68.2%
Treatment regimen		
Radiotherapy + surgery	61	36.9%
Surgery alone	25	16.2
Surgery + radiotherapy	68	44.2%
Type of tumors		
Lung carcinoma	35	22.7%
Renal-cell carcinoma	24	15.6%
Colon and rectal carcinoma	19	12.3%
Breast carcinoma	20	13.0%
Hepatocellular carcinoma	11	7.1%
Thyroid carcinoma	14	9.1%
Prostate carcinoma	10	6.5%
Uterus carcinoma	5	3.2%
Gastric carcinoma	3	1.9%
Bladder carcinoma	3	1.9%
Other rare and unknown carcinoma	10	6.5%
Bleeding control		
ABO*	80	51.9%
Without ABO	74	48.1%
Presenting signs and symptoms		
Motor	42	27.3%
Sensory	32	20.8%
Bladder	78	50.6%
Intestinal	76	49.4%
Pain	142	92.2%

*ABO = aortic balloon occlusion.

Medical records, histological slides, and image files were collected. Preoperative assessment included computerized tomography (CT) scanning, angiography, magnetic resonance imaging (MRI), whole body bone scintigraphy, or positron emission tomography (PET)–CT scan. The criteria for biopsy were as follows [9]: necessary for patients without a history of tumor but who were strongly suspected of metastases (10 cases); required for patients with a definite history of tumor in whom only one bony lesion was observed (64 cases); not required for patients with a definite history of tumor combined with multiple bony lesions (80 cases). The biopsies included 60 needle biopsies and 14 open biopsies. Based on Tomita scoring system, tumors were divided into three categories [10]. Breast ca, thyroid ca and prostatic ca belong to slow growth tumors. Renal cell ca, uterus ca, and colorectal ca belong to moderate growth tumors. Lung ca, gastric ca, hepatocellular ca, bladder ca and other rare and unknown ca belong to rapid growth tumors. A total of 129 patients with all types of tumors received radiotherapy at a dose of 30 Gy in 10 fractions, or 20 Gy in five fractions, or 24 Gy in six fractions as decided by the radiologists. Sixty-one patients received pre-operative radiotherapy and the mean period between radiotherapy and surgery was 7 months (3 to 20 months).

A single posterior approach was adopted for 145 patients and a combined posterior–anterior approach was adopted for nine patients before the implementation of aortic balloon occlusion (ABO) in 2003. Preoperative embolization was applied in 34 patients. Internal iliac artery ligation during an additional anterior approach or preoperative embolization was applied for the purpose of reducing intraoperative blood loss in cases of liver, kidney, and thyroid cancers, which have a rich blood supply. ABO was applied in 80 cases of tumors that invaded cephalad to the S2–S3 disc space, had a volume of > 200 cm^3^, or had an abundant blood supply [11]. The occlusion time was usually no more than 90 min. When a longer occlusion period was needed, the balloon was deflated for 10 min after the 90 min period and was then reinflated. The mean occlusion time was 86 min and the complications included femoral artery embolism in three patients and hematoma at the puncture site in one. The mean operative time was 177 min.

Intralesional curettage was used as the method for palliative surgery in all cases to minimize trauma and maximize functional recovery. Simple intralesional curettage was applied in 49 patients whose sacroiliac articulation was not invaded by metastatic lesions, while internal fixation (modified Galveston technique) after curettage was necessary in 105 patients whose sacroiliac articulation was invaded by metastatic lesions. Unlike for lumbar and thoracic vertebral bodies, bone cement cannot provide sufficient anchorage force in the sacral area and is also prone to compress the sacral nerve. Therefore, bone cement packing was not used in any patient.

Information about pre- and postoperative pain levels based on VAS scores and about bladder and bowel function was collected and assessed. Eleven potential prognostic factors were investigated [12]. Performance status was evaluated using the ECOG scale [13]. QoL was assessed using the European Organisation for Research and Treatment of Cancer core quality-of-life questionnaire (EORTC QLQ-C30) [14]. The QLQ-C30 was filled out by patients preoperatively and 3 months postoperatively when they came back to outpatient clinic for reexamination. Postoperative surgery complications including wound dehiscence, neurologic damage, cerebrospinal fluid leak, deep venous thrombosis, and internal fixation loosening were recorded. Routine follow-up evaluation was performed 3 months postoperatively and then every 3 months until local control failure (defined as reappearance of adverse symptoms or tumor recurrence in the region of surgery) or death. Each follow-up evaluation included clinical and neurologic examinations and imaging studies with standard radiographs, CT scan, and MRI. Patients suffering recurrence of a slow or moderate growth tumors or a radioresistant tumor were advised to undergo a second surgery. Those with rapidly growing or radiosensitive tumors were advised to undergo radiotherapy.

All statistical analyses were performed using SPSS software package version 16.0 (SPSS Inc., Chicago, IL, USA). The Kaplan–Meier method was used to estimate local control and survival rates. The log–rank test was used to compare the potential factors affecting local control. Multivariate analyses were performed using a Cox proportional hazards model. Paired *t* tests were used to assess the significance of the difference between the preoperative and 3-month postoperative results. A *p*-value < 0.05 was chosen to represent significance. Each domain of the QLQ-C30 was analyzed and presented separately because of its multidimensional method of assessment.

## Results

**Fig 1:**
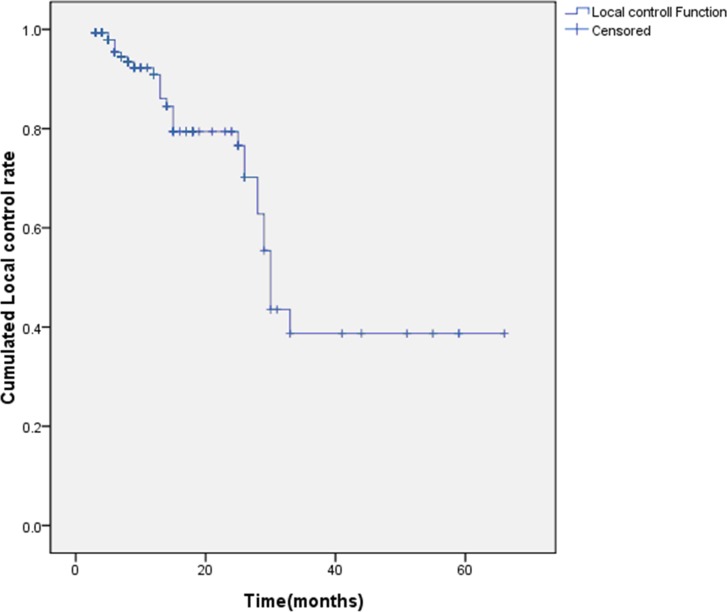
The cumulative rate of local control

The local control rate for the entire group was 95.4% at 6 months, 90.9% at 12 months, and 79.4% at 24 months (Fig 1). In univariate analysis, a rapidly growing tumors, absence of preoperative radiotherapy, and application of ABO were associated with good local control (Table 2). Multivariate analysis also indicated that these three factors were associated with a good local control rate.

**Table 2:** Univariate Analysis of Potential Prognostic Factors for Local Control Rate in the Study Participants.

Variable	No. of patients (%)	6-month survival (%)	1-year survival (%)	2-year survival (%)	*p*-value
Sex					
Male	87	96.0	89.7	83.9	0.262
Female	67	94.4	92.3	73.6	
Age (years)					
< 60	92	97.5	91.2	75.8	0.504
≥ 60	62	92.4	90.1	83.1	
Performance status					
0–2	110	94.9	90.4	76.0	0.351
3–4	44	96.4	91.8	91.8	
Neurological deficits					
No	51	92.5	87.4	81.5	0.895
Yes	103	96.8	92.4	78.8	
Tumor growth					
Rapid	62	97.5	92.6	92.6	<0.001
Moderate	48	97.8	97.8	97.8	
Slow	44	89.9	80.9	54.5	
Serum calcium level					
Normal	43	94.2	88.4	82.0	0.239
Elevated	111	95.8	91.6	78.5	
Visceral or cerebral metastases					
No	112	95.0	89.5	78.5	0.727
Yes	42	96.6	96.6	82.8	
Preoperative radiotherapy					
No	93	98.8	95.0	95.0	<0.001
Yes	61	90.0	84.2	49.5	
Disease-free interval					
No	74	98.5	93.7	87.5	0.484
Yes	80	92.5	88.3	70.6	
Multiple bony metastases					
No	64	96.2	88.9	76.3	0.619
Yes	90	94.8	92.4	82.3	
Aortic balloon occlusion					
No	74	89.7	82.5	57.3	<0.001
Yes	80	100	97.7	95.0	

Twenty-eight of the 154 patients (18.2%) experienced local control failure; seven received a second surgery, 10 received reirradiation, and 11 received systematic therapy such as chemotherapy and analgesic management. The cumulative survival rate of the entire group was 71.8% at 6 months, 41.1% at 12 months, and 22.5% at 24 months. The median overall survival for the entire cohort was 10 months (95% confidence interval [CI], 7.98–12.02 months).

There were 29 (18.8%) complications related to surgery, including 18 cases of poor wound healing, five cases of neurologic damage, four cases of cerebrospinal fluid leakage, one case of deep venous thrombosis, and one case of internal fixation loosening. Patients who underwent surgery without preoperative radiotherapy (n = 93) had a significantly decreased risk of postoperative complications compared with patients undergoing surgery after preoperative radiotherapy (n = 61) (*p* = 0.003, Pearson’s chi-square test). There were 11 and seven patients (total 29.5%) in the preoperative radiotherapy group who on admission developed complications of chronic radiation enteritis or cystitis, respectively.

All 142 patients suffer from intractable pain received opiate drugs with unsatisfied effect preoperatively while only 28 patients need analgesic intervention 3 months postoperatively. Sixty-one patients received radiotherapy and fifty patients received chemotherapy, targeted drugs or bisphosphonate for pain alleviation preoperatively. The mean VAS pain scores were 7.04 preoperatively, 1.66 at 1 month postoperatively (*p* = 0.003, paired *t* test), and 1.51 at 3 months postoperatively (*p* = 0.002, paired *t* test). The mean ECOG scores for performance status evaluation were 2.82 preoperatively and 1.47 at 3 months postoperatively (*p* < 0.001, paired *t* test). Postoperatively, 85 patients (55%) performed well (an ECOG status of 0 or 1) compared with 12 (7.8%) preoperatively. Improvement in performance was seen in 130 of the 154 patients (84.4%), while 21 patients (13.6%) remained unchanged and three (2.0%) deteriorated (Table 3). The mean functional and symptom scores on QLQ-C30 before surgery and at 3 months postoperatively are shown in Table 4. Paired *t* test analysis showed a significant improvement in scores for global QoL and symptom domains of pain and constipation from preoperatively to 3 months postoperatively. The metastatic lesions eroded L5/S1 nerve roots in 91 (59.1%) patients, which led to motor disability, while 37 (24.0%) patients had abnormal ambulation (walking with one assistive device or in a wheelchair) after surgery. Pre- and postoperative intestinal function changes are listed in Table 5, and the urinary function changes are listed in Table 6.

**Table 3:** Preoperative and 3-Month Postoperative Performance Status Evaluated by Eastern Cooperative Oncology Group (ECOG) Scoring System.

		3-month postoperative grade					
		0	1	2	3	4	Total
**Preoperative grade**	0						
	1	4	8				12
	2	5	35	5			45
	3	8	19	22	4	3	56
	4	1	5	28	3	4	41
	Total	18	67	55	7	7	154

**Table 4:** Quality of Life Evaluated by QLQ-C30 Before and After Surgical Treatment (mean, SD).

	Preoperative scores	Scores 3 months postoperatively	*p*-value
**Global quality of life**	43.3 (18.0)	52.1 (22.6)	0.000
**Functional scales**			
Physical	47.8 (15.6)	51.0 (14.1)	0.054
Role	51.9 (15.0)	54.9 (19.0)	0.062
Emotional	65.9 (17.8)	66.1 (18.3)	0.806
Cognitive	81.0 (10.6)	82.3 (11.3)	0.058
Social	47.1 (21.4)	49.0 (17.8)	0.097
**Symptom scale**			
Fatigue	39.4 (19.6)	38.6 (14.8)	0.254
Nausea and vomiting	13.6 (9.5)	13.0 (8.2)	0.279
Pain	61.1 (21.4)	32.5 (17.7)	0.000
Dyspnea	19.2 (16.1)	18.3 (13.5)	0.159
Sleep	32.9 (15.2)	31.1 (22.5)	0.067
Appetite loss	15.0 (12.4)	15.1 (13.6)	0.702
Constipation	50.9 (17.7)	28.8 (17.0)	0.000
Diarrhea	36.7 (13.4)	35.7 (14.2)	0.087
Financial difficulties	52.1 (10.5)	53.0 (13.5)	0.166

**Table 5:** Comparison of Pre- and Postoperative Intestinal Sphincter Muscle Function.

Bowel function	Normal	Abnormal but no	Medications/enemas	Colostomy,
		medication/enemas	required	diaper required
		required		
Preoperative	78	40	33	3
Postoperative	138	11	8	3

**Table 6:** Comparison of Pre- and Postoperative Urinary Sphincter Muscle Function.

Urinary function	Normal	Abnormal but	Intermittent	Permanent catheter
		no catheter	catheter	(total loss of
		required	required	bladder function)
Preoperative	78	46	22	8
Postoperative	136	8	7	3

## Discussion

With the emergence of more effective antineoplastic drugs and radiotherapy methods, patients with cancer survive longer. As the survival time has increased, more patients have acquired bony metastases: 5–10% of cancer patients develop spinal metastases during the course of their illness [15, 16]. Metastatic tumors comprise the majority of malignant lesions in the sacrum [17] but there is little information about outcomes for patients who accept surgical resection of sacral metastases, particularly in reports of large case series. Feiz-Erfan et al. reported the results of a study of 25 patients with sacral metastasis who underwent surgery, and summarized the indications of sacral metastasis [7]. However, the primary lesions in 15 of the 25 patients were renal carcinoma with rare pathologies. In addition, understanding how patients function after surgery and whether surgery for sacral metastasis can provide patients with a fine balance between local recurrence, survival, surgical complications, function, and overall QoL will help surgeons decide when to consider surgery and will make the significance of surgery clear [18]. This retrospective study is the first clinical study of a relatively large number of patients with sacral metastases that focuses on the effects of surgical intervention, including the oncological prognosis, surgical complications, clinical symptomatic improvement, and overall QoL.

This study has several limitations. The first is the lack of a control group of patients treated with radiotherapy alone; we are therefore unable to directly compare radiotherapy and surgery. We presume that patients underwent postoperative radiotherapy when the tumors were large and difficult to access in the sacrum or were sensitive to radiotherapy. Second, the best way to evaluate postoperative QoL and function is by dynamic analysis and follow-up at different periods not only including 3 months after surgery. However, owing to well-recognised difficulties in clinical follow-up, insufficient data were collected for analysis. Even though we were unable to analyze how function and QoL changed with time because of the difficulties in clinical data follow-up and collection, the overall influence of surgery on outcome at a particular postoperative time was still helpful when considering surgical treatment.

The indications for surgery include persistent pain, neurological impairment, tumors resistant to radiotherapy, or failure of local control after radiotherapy [19]. All the patients who underwent surgery in our study fulfilled the above indications and each patient had a Tomita score ≤ 7. Because cancers that have spread hematogenously to the sacrum are generally not curable, treatment efforts for sacral metastases are typically palliative, aiming at pain control and salvage of neurologic function [20]. Thus, the surgical margins were usually intralesional. Ozdemir et al. demonstrated that the posterior approach is preferable for tumors located distal to S3 while a combination of anterior and posterior approaches is recommended for tumors located at S1–S2 [1]. However, this conclusion was based on a complex series of primary and secondary malignant sacral tumors. A posterior approach alone was applied in most cases in our study, except for nine patients operated on before the introduction of ABO in 2003 who needed intraoperative bleeding control by surgical cross-clamping of the aorta, and who underwent a combination approach.

The rate of local control for our entire group was 95.4% at 6 months, 90.9% at 12 months, and 79.4% at 24 months. In a study by Mizumoto of patients with spinal metastases who received radiotherapy [11], the local control rates after 6, 12, and 24 months were 91% (95% CI, 88–94%), 79% (95% CI, 74–84%), and 69% (95% CI, 63–76%), respectively. Two other studies reported local control rates of respectively 84% and 71% as 1-year freedom from tumor progression [21, 22]. The comparatively low recurrence rate in the current study can be attributed to the adequate removal of tumor and effective hemorrhage control by ABO. To minimize tumor recurrence and to maximize the prevention of deterioration of neurologic function, or even to improve it, a dry and clear operation field is a prerequisite [23]. ABO mitigates significant risk factors for local control by providing effective hemorrhage control and thereby reduced blood loss, a clearer operation field, and adequate curettage of all suspicious areas [24]. Another significant risk factor, preoperative radiotherapy, may lead to a higher local recurrence rate because radiotherapy can cause scarring of both normal tissue and tumor, congestion, and severe adhesions, which may cause more hemorrhage and increase the difficulty for the surgeon to separate tumor from normal tissue, especially the sacral nerve. As a result, there is a high probability that tumor cells may adhere to normal tissue and be left in the operative region.

The cumulative survival rates of the entire group were 70.4%, 40.2%, and 22.3% at 6, 12, and 24 months, respectively, with a median survival time of 10 months, which were all lower than those reported in previous studies. For example, Nader et al. [8] reported overall survival rates of 75%, 61%, and 47% at 6, 12, and 24 months in patients who received surgery for sacral metastases, with a median survival time of 21.8 months. The lower survival rates in our study can be attributed to the higher proportion of rapidly growing tumors (62/154, 40.3%) than in the study by Nader et al. (2/19, 10.5%).

Traditionally, radiotherapy is chosen as the initial therapy for sacral metastasis. However, patients with spinal instability and neurological deterioration caused by bony compression of neural structures are unsuitable to undergo either traditional radiotherapy or the emerging form of radiotherapy called spinal stereotactic radiosurgery (SRS) [25–27]. In addition, patients receiving radiotherapy have a relatively shorter asymptomatic period than those undergoing surgery [28], and theoretically the rates of surgical complications and recurrence are higher when surgery is performed after failure of radiotherapy. In contrast, if patients receive surgery first, they still have the opportunity to receive radiotherapy immediately after surgery or upon tumor recurrence. Thus, we consider that surgery followed by radiotherapy is a more reasonable treatment for sacral metastases in a strictly selected patient group with spinal instability, neurological deterioration, or intractable pain caused by sacral nerve compression. Of course, the decision about treatment should be based on the patient’s wishes, and a multidisciplinary team approach should be applied to provide the best environment for establishing individualized management plans. Therefore, a randomized trial comparing the survival, local control, and complication rates of radiotherapy alone with those of radiotherapy plus surgery and with surgery plus postoperative radiotherapy is recommended.

Li et al. reported that preoperative radiotherapy is an independent risk factor for wound complications after surgery for sacral tumors and suggested that late vascular changes after radiotherapy may adversely affect wound healing [17, 29–31]. We saw a strong correlation between surgical complications and the implementation of surgery combined with preoperative radiotherapy (Table 7; Pearson’s chi-square test, *p* = 0.006). Neurologic damage and cerebrospinal fluid leakage were attributed to severe adhesions between the tumor and the sacral nerve dura or even the sacral nerve fiber after radiotherapy.

**Table 7:** Correlation of Preoperative Radiotherapy with Surgical Complications.

	With complications	Without complications	Total
With preoperative radiotherapy	18	43	61
Without preoperative radiotherapy	11	82	93
Total	29	125	

Radiotherapy not only increases the complication rate of sacral metastasis surgery, but also itself produces some severe complications. Of patients undergoing radiation to the pelvis, 50–75% experience symptoms of acute radiation proctitis [32, 33], and the incidence of chronic radiation proctitis symptoms can be as high as 30% [34, 35]. There are also reports that 5–15% of the patients who receive abdominal or pelvic irradiation will develop chronic radiation enteritis. The overall frequency of radiation cystitis 1 year after treatment of bladder cancer is 9–21% [36]. In our study, 11 and 7 patients (total 29.5%) in the preoperative radiotherapy group developed complications of chronic radiation enteritis and cystitis, respectively. Li et al. [29] demonstrated that the specific method applied to reduce intraoperative blood loss (135 of 183 patients underwent ABO) was a risk factor for wound complications in univariate analysis but was not significant in multivariate analysis. We found no correlation in our study between the application of ABO and complications (Pearson’s chi-square test, *p* = 0.113).

Several instruments are used for global QoL assessment in patients with cancer, most of which are designed either for the general population or for patients undergoing treatment for tumors [37]. The QLQ-C30 is a validated method for evaluating health-related QoL in patients with cancer [38]. Wu et al. [39] reported an improvement in total QoL, physical well-being, emotion, and function for patients with spinal metastases. Piccioli et al. reported an improvement in the general condition of patients with pathological femoral fracture using QOL-ACD [40]. Ji et al. reported significant changes in global QoL and pain assessed by QLQ-C30 for patients with periacetabular metastases; they suggested that pain has a central importance in a patient’s overall assessment of QoL and predicted that an improvement in the control of pain would result in an improvement in QoL [18]. In our study, we found significant improvement in global QoL, pain, and constipation after surgery. Surgery eliminated the tumor compressing the sacral nerve, which rapidly and effectively relieved pain and constipation. According to the theory of Ji et al., global QoL was improved as a result of pain relief.

Despite the limitations of our study, the results clearly show that in patients with metastatic involvement of the sacrum, surgical intervention offers benefits in terms of pain relief and improvements in constipation, ECOG, and QoL with a low rate of complications.

## Supporting Information

S1 File Strobe Statement (DOCX)

S2 File Primary data (XLSX)
