# Supplementary material for: What Is the Value of Surgical Intervention for Sacral Metastases?
Source: PLoS One. 2016 Dec 19;11(12):e0168313. doi: 10.1371/journal.pone.0168313 (PMC5167270; doi:10.1371/journal.pone.0168313)
Supplement: S1 File — (DOCX) [file pone.0168313.s001.docx]

STROBE Statement—checklist of items that should be included in reports of observational studies

|  | Item No. | Recommendation | Page  No. | | Relevant text from manuscript |
| --- | --- | --- | --- | --- | --- |
| **Title and abstract** | 1 | (*a*) Indicate the study’s design with a commonly used term in the title or the abstract | 1 | | surgical intervention for sacral metastases |
|  |  | (*b*) Provide in the abstract an informative and balanced summary of what was done and what was found | 1 | | Surgery for sacral metastasis is effective to palliate pain rapidly, improve constipation and quality of life with low rate of complications |
| Introduction | | | | |  |
| Background/rationale | 2 | Explain the scientific background and rationale for the investigation being reported | 1-2 | | Metastatic bone disease affecting the sacrum is increasingly encountered by oncologists and orthopaedic surgeons. The effects and complications of surgery for sacral metastases were still unclear. |
| Objectives | 3 | State specific objectives, including any prespecified hypotheses | 2 | | To detected the impact of surgery on local control, improvement of VAS, ECOG and QoL for sacral metastases and whether the complications of surgery were acceptable. |
| Methods | | | | |  |
| Study design | 4 | Present key elements of study design early in the paper | | 7 | Information of pre and postoperative pain levels based on a visual analog scale (VAS), function of bladder and bowel were collected and assessed. Eleven potentially prognostic factors were investigated [11]. Performance status was evaluated using the Eastern Cooperative Oncology Group (ECOG) performance status scale [12]. Quality of life was assessed using the European Organisation for Research and Treatment of Cancer core quality of-life questionnaire (EORTC QLQ-C30) [13]. The postoperative surgery complications were recorded including wound dehiscence, neurologic damage cases, CSF leak, deep venous thrombosis and internal fixation loosening. |
| Setting | 5 | Describe the setting, locations, and relevant dates, including periods of recruitment, exposure, follow-up, and data collection | | 3 | The records of 180 patients with sacral metastases admitted in Musculoskeletal Tumor Centre of Peking University People's Hospital, China between July 1997 and June 2015 were reviewed retrospectively. |
| Participants | 6 | (*a*) *Cohort study*—Give the eligibility criteria, and the sources and methods of selection of participants. Describe methods of follow-up  *Case-control study*—Give the eligibility criteria, and the sources and methods of case ascertainment and control selection. Give the rationale for the choice of cases and controls  *Cross-sectional study*—Give the eligibility criteria, and the sources and methods of selection of participants | | 4 | The inclusive criteria included diagnosed metastatic sacral tumor, patients received surgical intervention and patients received complete follow-up for at least 3 months. We excluded 10 patients who refused to accepted surgery modality, 8 patients who lost to follow-up, 6 patients who received en-bloc resection with lesions located below S3 and 2 patients who died early postoperatively from complications not associated with the primary tumor. Routine follow-up evaluation was performed 3 month and every 3 months post-operatively until local control failure (defined as adverse symptoms reappearance or tumor recurrence in the surgery region) or death. |
|  |  | (*b*)*Cohort study*—For matched studies, give matching criteria and number of exposed and unexposed  *Case-control study*—For matched studies, give matching criteria and the number of controls per case | |  |  |
| Variables | 7 | Clearly define all outcomes, exposures, predictors, potential confounders, and effect modifiers. Give diagnostic criteria, if applicable | | 7 | Information of pre and postoperative pain levels based on a visual analog scale (VAS), function of bladder and bowel were collected and assessed. Eleven potentially prognostic factors were investigated. Performance status was evaluated using the Eastern Cooperative Oncology Group (ECOG) performance status scale . Quality of life was assessed using the European Organisation for Research and Treatment of Cancer core quality of-life questionnaire (EORTC QLQ-C30) . The postoperative surgery complications were recorded including wound dehiscence, neurologic damage cases, CSF leak, deep venous thrombosis and internal fixation loosening. |
| Data sources/measurement | 8* | For each variable of interest, give sources of data and details of methods of assessment (measurement). Describe comparability of assessment methods if there is more than one group | | 10 | Table 2 |
| Bias | 9 | Describe any efforts to address potential sources of bias | |  |  |
| Study size | 10 | Explain how the study size was arrived at | |  |  |

Continued on next page

| Quantitative variables | 11 | Explain how quantitative variables were handled in the analyses. If applicable, describe which groupings were chosen and why | 8 | The Kaplan-Meier approach was used to estimate local control and survival rate. The log-rank test was used to compare the potential factors of local control. Multivariate analyses were performed using a Cox proportional hazard model. Paired *t*-tests were used to assess the significance of the difference between the pre-operative and 3 month postoperatively. A p-value < 0.05 was chosen to represent significance. Each domain of the QLQ-C30 was analyzed and presented separately because of its multidimensional method of assessment. |
| --- | --- | --- | --- | --- |
| Statistical methods | 12 | (*a*) Describe all statistical methods, including those used to control for confounding |  |  |
|  |  | (*b*) Describe any methods used to examine subgroups and interactions |  |  |
|  |  | (*c*) Explain how missing data were addressed |  |  |
|  |  | (*d*) *Cohort study*—If applicable, explain how loss to follow-up was addressed  *Case-control study*—If applicable, explain how matching of cases and controls was addressed  *Cross-sectional study*—If applicable, describe analytical methods taking account of sampling strategy | 4 | 8 patients who lost to follow-up were excluded |
|  |  | (*e*) Describe any sensitivity analyses |  |  |
| Results | | | | |
| Participants | 13* | (a) Report numbers of individuals at each stage of study—eg numbers potentially eligible, examined for eligibility, confirmed eligible, included in the study, completing follow-up, and analysed | 4 | Table 1 |
|  |  | (b) Give reasons for non-participation at each stage |  | N/A |
|  |  | (c) Consider use of a flow diagram |  | N/A |
| Descriptive data | 14* | (a) Give characteristics of study participants (eg demographic, clinical, social) and information on exposures and potential confounders | 10 | Table 2 |
|  |  | (b) Indicate number of participants with missing data for each variable of interest | 4 | 8 patients who lost to follow-up were excluded |
|  |  | (c) *Cohort study*—Summarise follow-up time (eg, average and total amount) | 7 | Routine follow-up evaluation was performed 3 month and every 3 months post-operatively until local control failure (defined as adverse symptoms reappearance or tumor recurrence in the surgery region) or death. |
| Outcome data | 15* | *Cohort study*—Report numbers of outcome events or summary measures over time |  |  |
|  |  | *Case-control study—*Report numbers in each exposure category, or summary measures of exposure |  |  |
|  |  | *Cross-sectional study—*Report numbers of outcome events or summary measures |  |  |
| Main results | 16 | (*a*) Give unadjusted estimates and, if applicable, confounder-adjusted estimates and their precision (eg, 95% confidence interval). Make clear which confounders were adjusted for and why they were included | 12 | There were 29(18.8%) complications related to surgery, including 18 poor wound healing cases, 5 neurologic damage cases, 4 CSF leak cases, 1 deep venous thrombosis cases and 1 internal fixation loosening case. Patients who underwent surgery without preoperative radiotherapy (n=93) had a significant decreased risk of post-operative complications compared with patients undergoing surgery after pre-operative radiotherapy (n=61) (p =0.006; Pearson Chi-Square test). There are 11 and 7 patients (totally 29.5%) in the preoperative radiotherapy group developed complications of chronic radiation enteritis and cystitis respectively on admission. |
|  |  | (*b*) Report category boundaries when continuous variables were categorized |  |  |
|  |  | (*c*) If relevant, consider translating estimates of relative risk into absolute risk for a meaningful time period |  |  |

Continued on next page

| Other analyses | 17 | Report other analyses done—eg analyses of subgroups and interactions, and sensitivity analyses |  | N/A |
| --- | --- | --- | --- | --- |
| Discussion | | | | |
| Key results | 18 | Summarise key results with reference to study objectives | 21 | Despite our study has limitations, it clearly shows that in patients with metastatic  involvement of the sacrum, surgical intervention offers benefits in terms of pain relief, constipation, ECOG and QoL improvement with an low rate of complications. |
| Limitations | 19 | Discuss limitations of the study, taking into account sources of potential bias or imprecision. Discuss both direction and magnitude of any potential bias | 16 | First is the lack of a control group of patients treated by radiotherapy alone.  Second, the best way to evaluate post-operative function is by dynamic analysis and follow-up at different period after surgery. |
| Interpretation | 20 | Give a cautious overall interpretation of results considering objectives, limitations, multiplicity of analyses, results from similar studies, and other relevant evidence | 8 | Multivariate analysis also indicated that these three factors were associated with good local control rate. |
| Generalisability | 21 | Discuss the generalisability (external validity) of the study results |  | N/A |
| Other information | |  | | |
| Funding | 22 | Give the source of funding and the role of the funders for the present study and, if applicable, for the original study on which the present article is based | 21 | The authors did not receive any outside funding or grants in support of their research for or preparation of this work. Neither they nor a member of their immediate families received payments or other benefits or a commitment or agreement to provide such benefits from a commercial entity. |

*Give information separately for cases and controls in case-control studies and, if applicable, for exposed and unexposed groups in cohort and cross-sectional studies.

**Note:** An Explanation and Elaboration article discusses each checklist item and gives methodological background and published examples of transparent reporting. The STROBE checklist is best used in conjunction with this article (freely available on the Web sites of PLoS Medicine at http://www.plosmedicine.org/, Annals of Internal Medicine at http://www.annals.org/, and Epidemiology at http://www.epidem.com/). Information on the STROBE Initiative is available at www.strobe-statement.org.
